# Antinociceptive and Anti-Inflammatory Activities of Leaf Methanol Extract of *Cotyledon orbiculata* L. (Crassulaceae)

**DOI:** 10.1155/2012/862625

**Published:** 2011-11-16

**Authors:** George J. Amabeoku, Joseph Kabatende

**Affiliations:** Discipline of Pharmacology, School of Pharmacy, University of the Western Cape, Private Bag X17, Bellville 7535, South Africa

## Abstract

Leaf methanol extract of *C. orbiculata* L. was investigated for antinociceptive and anti-inflammatory activities using acetic acid writhing and hot-plate tests and carrageenan-induced oedema test in mice and rats, respectively. *C. orbiculata* (100–400 mg/kg, i.p.) significantly inhibited acetic acid-induced writhing and significantly delayed the reaction time of mice to the hot-plate-induced thermal stimulation. Paracetamol (300 mg/kg, i.p.) significantly inhibited the acetic acid-induced writhing in mice. Morphine (10 mg/kg, i.p.) significantly delayed the reaction time of mice to the thermal stimulation produced with hot plate. Leaf methanol extract of *C. orbiculata* (50–400 mg/kg, i.p.) significantly attenuated the carrageenan-induced rat paw oedema. Indomethacin (10 mg/kg, p.o.) also significantly attenuated the carrageenan-induced rat paw oedema. The LD_50_ value obtained for the plant species was greater than 4000 mg/kg (p.o.). The data obtained indicate that *C. orbiculata* has antinociceptive and anti-inflammatory activities, justifying the folklore use of the plant species by traditional medicine practitioners in the treatment of painful and inflammatory conditions. The relatively high LD_50_ obtained shows that *C. orbiculata* may be safe in or nontoxic to mice.

## 1. Introduction

Pain and inflammation are some of the most common manifestations of many diseases afflicting millions of people worldwide [1, 2]. Even though there are effective orthodox medicines used to alleviate these manifestations [3], traditional medicine practitioners in, mainly, developing countries have used herbal medicines to treat various ailments including pain and inflammation [4]. The dependence of the population especially in the rural communities in South Africa on plant medicines as well as traditional medicine practitioners for their healthcare needs is cultural. One of such plants used by traditional medicine practitioners to treat various ailments is *Cotyledon orbiculata* L. [5, 6]. It belongs to the family Crassulaceae. It is a small shrub with fleshy leaves and widely distributed in Southern Africa. It is known locally as “Seredile” in Sotho and Tswana, “Plakkie” in Afrikaans, and “Imphewula” in Xhosa [5, 6]. *C. orbiculata* is used in the treatment of various ailments in different parts of South Africa. The fleshy leaves have been used to treat corn and warts. The juice of the leaves is used as drops for earache and toothache and as hot poultice for boils and inflammation [5–7]. Infusion of the fleshy leaves of *C. orbiculata* has also been used by traditional medicines practitioners in South Africa for the treatment of epilepsy, inflammation, and aches (Oral communication).

According to the literature, very limited evaluation has been done on the pharmacological activities of the plant species despite the wide folklore use [8]. This study was, therefore, intended to investigate the antinociceptive and anti-inflammatory activities of *C. orbiculata* in mice and rats, respectively. The acute toxicity and HPLC studies of the plant species were also carried out.

## 2. Materials and Methods

### 2.1. Plant Material

The fleshy leaves of *C. orbiculata* were collected from Kirstenbosch National Botanical Garden, Cape Town, in September, 2010. The plant material was identified by the curator of the Gardens as well as a taxonomist in the Department of Biodiversity and Conservative Biology, University of the Western Cape and the voucher specimen (COT 25) deposited in the University's Herbarium.

### 2.2. Preparation of Plant Extract

The fleshy leaves (10.5 kg) of *C. orbiculata *were washed with water, sliced into pieces, and dried in a ventilated oven at 40°C for 120 h. The dried plant material (640 g) was ground into fine powder using Waring Commercial laboratory blender and passed through 850 *μ*m sieve. For the preparation of the methanol extract, the dried powder (120 g) was extracted in a soxhlet extractor with methanol for 72 h. The methanol filtrate was evaporated to dryness using a Buchi RE11 rotavapor and Buchi 461water bath. A yield of 55.4 g of crude methanol extract was obtained and preserved in a dessicator. Fresh solution of the crude leaf methanol extract was prepared by dissolving a given quantity of the methanol extract in a small volume of dimethylsulfoxide (DMSO) and made up to the appropriate volume with physiological saline. The methanol solution was administered intraperitoneally (i.p.) to mice and rats in a volume of 1 mL/100 g of body weight.

### 2.3. Animals

Male albino mice bred in the Animal House of the Discipline of Pharmacology, School of Pharmacy, University of the Western Cape, South Africa, weighing 18–30 g were used for the antinociceptive activity and acute toxicity studies. Young adult male Wistar rats, bought from the University of Cape Town, South Africa, and weighing 160–210 g were used for anti-inflammatory activity study. The animals were housed in a quiet laboratory with an ambient temperature of 22 ± 1°C and a12 h light/12 h dark cycle was maintained. They all had access to food and water *ad libitum*. All the animals were fasted for 16 h during which they had access to water prior to the commencement of the experiments. Each animal was used for one experiment only.

### 2.4. Drugs and Chemicals

Indomethacin (Sigma Chemical Co.) was dissolved in a minimum amount of dimethylsulfoxide (DMSO, Sigma Chemical Co.) and adjusted to the appropriate volume with physiological saline. Carrageenan (Sigma Chemical Co.) and morphine sulphate (Bodene) were dissolved in physiological saline to an appropriate volume. Acetic acid (Merck) was dissolved in physiological saline to an appropriate strength. Paracetamol (Sigma Chemical Co.) was dissolved in a minimum volume of propylene glycol 400 (BDH, UK) and adjusted to the appropriate volume with physiological saline. DMSO solution was prepared by dissolving an equal amount of DMSO used to dissolve the plant extract, in an appropriate volume of physiological saline. Indomethacin was given orally to rats by means of a bulbed steel needle. Carrageenan was injected into the subplantar surface of the right hind paws of the rats.

Morphine, acetic acid, and paracetamol were administered intraperitoneally (ip) to mice. Fresh drug solutions were prepared each morning of the experiment. All drugs were administered in a volume of 1 mL/100 g of body weight, while constant volumes of carrageenan, DMSO, physiological saline, and acetic acid were used. Control animals received equal volume injections of the appropriate vehicles. The doses and pretreatment times of the leaf methanol extract of *C. orbiculata* and standard drugs, indomethacin, morphine, paracetamol, and the vehicles, physiological saline and DMSO, were obtained from preliminary studies in our laboratory.

## 3. Assessment Pharmacological Activities

### 3.1. Antinociceptive Activity of *Cotyledon orbiculata*

#### 3.1.1. Acetic Acid Writhing Test

The methods of Koster et al. [9] and Williamson et al. [10] were used for the assessment of the antinociceptive activity of *C. orbiculata*. Mice were used in groups of 8 per dose of plant extract, standard drug, paracetamol, or DMSO. They were placed singly in a transparent perspex mouse cage and allowed to acclimatize to their environment for 30 min prior to the commencement of the experiment. In the control experiment, the animals were pretreated with 0.25 mL of physiological saline (i.p.) for 15 min and then given intraperitoneal injection of 0.20 mL of 3% acetic acid solution, an irritant, used to induce writhing (pain). The mice were then left for 5 min, and the writhes were counted for the next 20 min. A writhe is defined as contraction of the abdominal muscles accompanied by elongation of the body and the hind limbs.

In the test experiment, a group of 8 mice were pretreated for 15 min with either the plant extract (i.p.) or the standard analgesic drug, paracetamol (i.p.), after which they were injected with 0.20 mL of the 3% acetic acid intraperitoneally, allowed to stand for 5 min and then the number of writhes counted for 20 min as for the control experiment. The experiment was repeated with another group of 8 mice pretreated with 0.25 mL of DMSO solution (i.p.) for 15 min, after which they were injected with 0.20 mL of the 3% acetic acid intraperitoneally, allowed to stand for 5 min, and then the number of writhes counted for 20 min. All experiments were performed in a quite laboratory with an ambient temperature of 22 ± 1°C. The ability of the plant extract to prevent or significantly reduced the number of acetic acid-induced writhes was an indication of an antinociceptive activity.

#### 3.1.2. Hot-Plate Test

The methods of Williamson et al. [10] and Eddy and Leimback [11] were used in the hot-plate test for the antinociceptive activity of *C. orbiculata*. Mice were used in groups of 8 per dose of plant extract, standard drug, morphine, or DMSO. Control animals were individually placed in a 21 glass beaker placed on a thermostatically controlled hot plate (model HC500, Bibby Sterilin Ltd., England) set at 50–55°C, before and 15 min after intraperitoneal injection of 0.25 mL of physiological saline. The pain threshold is considered to be reached when the animals lift and lick their paws or attempt to jump out of the beaker. The time taken for the mice to exhibit these characteristics, also known as the reaction or response time, was noted by means of a stopwatch. The animals were tested before and 15 min, 30 min, 45 min, and 60 min after intraperitoneal injection of 0.25 mL of physiological saline. The experiments were repeated using other groups of animals, which were tested before and 15 min, 30 min, 45 min, and 60 min after the intraperitoneal administration of either the plant extract, morphine, or DMSO. All experiments were performed in a quite laboratory with an ambient temperature of 22 ± 1°C. A cutoff time of 60 s was used to avoid harm to the mice. The ability of the plant extract to delay the reaction time was taken as an indication of an antinociceptive activity.

### 3.2. Anti-Inflammatory Activity of *Cotyledon orbiculata*

#### 3.2.1. Rat Paw Oedema Test

Modified method of Williamson et al. [10] and Winter et al. [12] were used to assess the anti-inflammatory activity of *C. orbiculata*. Rats were used in groups of 8 per dose of plant extract, standard drug, physiological saline, or DMSO. The rats were divided into five groups. Rats in Group I (control) were given 0.25 mL (i.p.) of physiological saline. Group II rats received plant extracts (50–400 mg/kg, i.p.). Group III rats were given the standard anti-inflammatory drug, indomethacin (10 mg/kg, p.o.), and Group IV rats received 0.25 mL (i.p.) of DMSO (vehicle). Group V rats were untreated. Oedema or acute inflammation was induced in Group I or control rats pretreated for 15 min with 0.25 mL (i.p.) of physiological saline by injecting 0.1 mL of carrageenan (1% dissolved in 0.9% saline solution) into the subplantar surface of the right hind paw. The oedema following the carrageenan injection was noticeable within 30–40 min. The volume of the right hind paw was measured before and then after the injection of carrageenan at 30 min intervals for 4 h by volume displacement method using plethysmometer (IITC Life Sciences, USA). Group II rats were pretreated for 15 min with plant extracts intraperitoneally (i.p.), Group II rats for 1 h with indomethacin orally (p.o.) and Group IV rats for 15 min with DMSO (i.p.) prior to the injection 0.1 mL of carrageenan into the subplantar surface of the right hind paws of the rats in each group. The experiments were repeated with the volumes of the rats' right hind paws measured before and then after the injection of carrageenan at 30 min intervals for 4 h using the plethysmometer. The volumes of the untreated rats' right paws were also measured at 30 min intervals for 4 h. Oedema was expressed as a mean increase in paw volume with respect to physiological saline control. Inhibition was expressed as a percentage increase or decrease in oedema volume. The ability of the plant extract to inhibit the foot oedema was taken as an indication of an anti-inflammatory activity. All experiments were performed in a quite laboratory with an ambient temperature of 22 ± 1°C.

#### 3.2.2. HPLC Analysis

Chromatographic system: Beckman HPLC system consisting of double pump Programmable Solvent Module model 126; Diode Array detector Module model 168; Samsung computer 386 with management System Gold (Gold V601) software supplied by Beckman; Column, C18 Bondapak 5 *μ*m and dimensions (250 × 4.6 mm).

Chromatographic conditions: Mobile phase: solvent A: 1% acetic acid; solvent B: methanol; Mode: gradient; flow rate, 1 min/min; injection volume, 10 *μ*L; detector, UV at 350 nm. The HPLC operating conditions were programmed to give the following: 0 min, solvent B: 20%; 5 min, solvent B: 40%; 15 min, solvent B: 60%; 20 min, solvent B: 80% and 27 min, solvent B: 20%. The run rate was 30 min.

#### 3.2.3. Acute Toxicity Testing

The method described by Lorke [13] and modified by Hilaly et al. [14] was used to determine the median lethal dose (LD_50_) of the leaf methanol extract. Mice were fasted for 16 h and then randomly divided into groups of eight mice per cage. Graded doses of the plant extract (100, 200, 300, 400, 600, 800, 1600, 2000, 2400, 2800, 3200, 3600, and 4000 mg/kg) were separately administered orally by means of a bulbed steel needle to mice in each test group. The control group was administered with 0.25 mL (p.o.) of physiological saline by means of a bulbed steel needle. The mice in both the test and control groups were then allowed free access to food and water and observed for over 5 days for signs of acute toxicity including death. The median lethal dose (LD_50_) of the leaf methanol extract of *C. orbiculata* would be calculated if applicable, from a plot of log dose-response curve which would be constructed for the plant species.

### 3.3. Statistical Analysis

The data on the number of writhes exhibited by the mice and the effect of carrageenan on the rat's right hind paw were analysed using one way analysis of variance (ANOVA) followed by Dunnett's multiple comparison test (GraphPad Prism, version 5.0, GraphPad Software, Inc., SanDiego CA p2130, USA) and presented as mean ± standard error mean (SEM). *P* values of less than 5% (*P* < 0.05) were considered statistically significant.

### 3.4. Ethical Considerations

The experimental protocol used in this study was approved (07/04/31) by the Ethics Committee of the University of the Western Cape, Bellville 7535, South Africa, and conforms with the University's Regulations Act concerning animal experiments.

## 4. Results

### 4.1. Pharmacological Activities: Antinociceptive Activity of *Cotyledon orbiculata*

#### 4.1.1. Acetic Acid Writhing Test

**Effect of Leaf Methanol Extract of *Cotyledon orbiculata* on Acetic Acid-Induced Writhing**

0.20 mL (i.p.) of 3% acetic acid produced a substantial number of writhes in control mice pretreated with 0.25 mL (i.p.) of physiological saline. Leaf methanol extract of *C. orbiculata* (100–400 mg/kg, i.p.) in a dose-dependent manner, significantly reduced the number of acetic acid-induced writhes. 100 mg/kg (i.p.) of the plant species reduced the writhes by 51%. 200 mg/kg (i.p.) and 400 mg/kg (i.p.) of *C. orbiculata* produced 67% and 76% reduction in writhes produced by 0.20 mL of 3% acetic acid in mice, respectively. Similarly, paracetamol (300 mg/kg, i.p.) profoundly reduced the number of writhes elicited by 0.20 mL of 3% acetic acid by 93%. DMSO (0.25 mL, i.p.) did not significantly alter the acetic acid-induced writhes in mice (Table 1).

**Effect of Leaf Methanol Extract of *Cotyledon orbiculata* on Hot-Plate-Induced Nociception**

Mice pretreated with physiological saline reacted to hot-plate thermal stimulation at 50°C–55°C either by lifting and licking their paws or attempting to jump out of the beaker. This manifestation occurred within 6.63 ± 0.60 sec in the first 15 min after intraperitoneal administration of 0.25 mL of physiological. saline and within 2.75 ± 0.31 sec, 60 min later after the injection of 0.25 mL of physiological saline. Leaf methanol extract of *C. orbiculata* (100–200 mg/kg, i.p.) significantly delayed the reaction times of the animals to hot-plate thermal stimulation 30 min after treatment.* C. orbiculata* (400 mg/kg, i.p.) significantly delayed the pain reaction time of the mice to the hot-plate-induced thermal stimulation over the 1 h period of measurement. Similarly, morphine (10 mg/kg, i.p.) significantly delayed the reaction time of the mice to the hot-plate-induced thermal stimulation over the 1 h period of measurement. DMSO (0.25 mL, i.p.) did not significantly alter the reaction time of the mice to the hot-plate-induced thermal stimulation over the 1 h period of measurement (Table 2).

**Effect of Leaf Methanol Extract of *C. orbiculata* on Carrageenan-Induced Right Hind Paw Oedema**

Carrageenan (1%) injected into the subplantar of the right hind paws of the rats pretreated with physiological saline induced oedema or acute inflammation in the paws within 30–40 min. The oedema reached its maximum intensity 3 h after injection. 50 mg/kg (i.p.) of the leaf methanol extract of *C. orbiculata* significantly reduced the carrageenan-induced oedema from 60 min up to the 4 h period of measurement. *C. orbiculata* (100–400 mg/kg, i.p.) significantly reduced the carrageenan-induced oedema over the 4 h period of measurement. Indomethacin (10 mg/kg, p.o.) profoundly reduced the carrageenan-induced oedema in the right hind paws of rats over the 4 h period of measurement (Table 3).

#### 4.1.2. Acute Toxicity Test

There were no deaths or signs of acute toxicity observed after oral administration of 100–4000 mg/kg of the leaf methanol extract of *Cotyledon orbiculata *with the highest dose tested (4000 mg/kg, p.o.) being the no-adverse-effect-level (NOAEL). That is, the LD_50_ was probably greater than 4000 mg/kg (p.o.) in mice.

#### 4.1.3. HPLC Analysis

**Figure 1:**
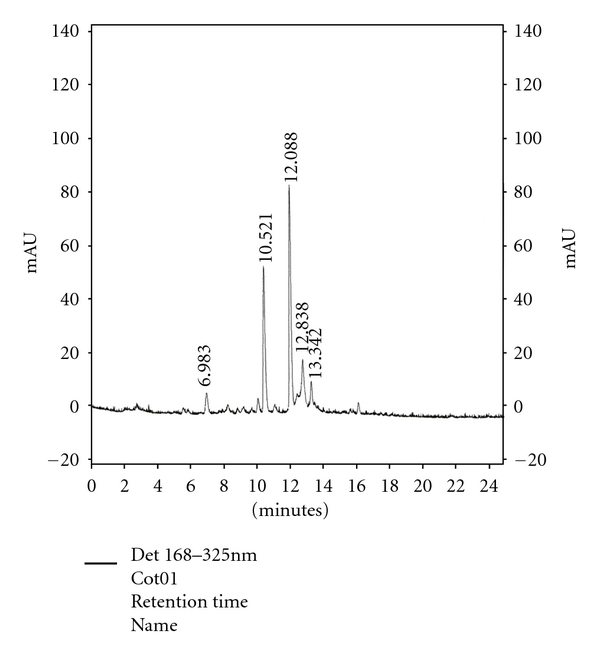
HPLC fingerprint of leaf methanol extract of *Cotyledon orbiculata. *

The chromatographic spectrum of the leaf methanol extract of *C. orbiculata* obtained revealed major peaks at the following retention times (minutes): 6.983, 10.521, 12.088, 12.838, and 13.342 (Figure 1).

## 5. Discussion

In the present study, the leaf methanol extract of *C. orbiculata* significantly inhibited the acetic acid-induced writhing and significantly inhibited the nociception produced by hot plate. *C. orbiculata* also significantly attenuated carrageenan-induced rat right hind paw oedema. Satyanarayana et al. [15] has shown that acetic acid produced writhing or nociception by stimulating the production of prostaglandin. Paracetamol, a standard analgesic drug [2], has been shown to inhibit prostaglandin synthesis in the brain [16]. It is, therefore, not surprising that paracetamol significantly attenuated acetic acid-induced nociception in this study. The effect of paracetamol on prostaglandin in relation to acetic-acid-induced writhes may be direct or indirect. Since *C. orbiculata* also attenuated acetic acid-induced writhing, it is probable that the plant species may be producing its antinociceptive activity by affecting the prostaglandin system. Morphine, a standard centrally acting analgesic drug [3], significantly attenuated the thermal stimulation or nociception produced by the hot plate. *C. orbiculata* also significantly attenuated the nociception produced by hot plate. It is probable that the plant species may be acting via certain central pain receptors to attenuate the nociception produced by hot plate in this study. According to Koster et al. [9], Williamson et al. [10] and Eddy and Leimback [11], acetic acid writhing and hot plate tests are used to evaluate peripherally and centrally acting analgesic drugs respectively. In this study, *C. orbiculata* attenuated both the acetic acid-induced writhing and the nociception produced by hot plate which may suggest that the plant species may have both peripheral and central antinociceptive effect.

Swingle [17] has shown that prostaglandins, histamine, serotonin, and bradykinin are mediators of different phases of carrageenan-induced oedema. Di Rosa et al. [18], Capasso et al. [19], and Salvemini et al. [20] have also reported the involvement of histamine, 5-hydroxytrptamine, bradykinin, prostaglandin, and nitric oxide in carrageenan-induced paw oedema. Nag-Chaudhuri et al. [21] in their report on their work on the anti-inflammatory and related actions of *Syzygium cuminii* seed extract suggested that prostaglandin E_1_, histamine, serotonin, and bradykinin mediate carrageenan-induced rat paw oedema. Indomethacin has been shown to produce its anti-inflammatory effect by inhibiting the enzyme, cyclooxygenase, thus inhibiting prostaglandin synthesis [22]. It has also been shown that the nonsteroidal anti-inflammatory drugs may antagonize mediators such as serotonin, bradykinin, and capsaicin [23] some of which have been implicated in carrageenan-induced paw oedema. It is not surprising that in this study, indomethacin attenuated carrageenan-induced rat right hind paw oedema. *C. orbiculata* also attenuated the carrageenan-induced rat right hind paw oedema which may suggest that probably, the plant species may be affecting a host of mediators to produce its anti-inflammatory effect.

Amabeoku et al. [8] have shown that the leaves of *C. orbiculata* contain tannins, saponins, triterpene steroid, reducing sugar, and cardiac glycosides. Bruneton [24] reported that saponins have both analgesic and anti-inflammatory properties. It is possible, therefore, that saponins may also be contributing to the antinociceptive and anti-inflammatory activities of *C. orbiculata* in this study. The HPLC fingerprint of the plant species obtained revealed major characteristic peaks at the following retention times (minutes): 6.983, 10.521, 12.088, 12.838, and13.342. The acute toxicity test carried out showed that the LD_50_ value obtained for *C. orbiculata* could be greater than 4000 mg/kg (p.o.).

In conclusion, the data obtained show that *C. orbiculata* has both antinociceptive and anti-inflammatory activities which may be produced by the plant species inhibiting various chemical mediators including prostaglandins and bradykinin. The relatively high LD_50_ value of 4000 mg/kg (p.o.) obtained for the plant species shows that it may be safe in or nontoxic to mice. The result obtained justifies the use of the plant species by traditional medicine practitioners in South Africa for the treatment of painful conditions such as headache, earache, toothache, and inflammation. However, more studies are needed to further elucidate the mechanism of the antinociceptive and anti-inflammatory actions of *C. orbiculata*.

## Figures and Tables

**Table 1:** Effect of leaf methanol extract of *Cotyledon orbiculata* on acetic acid-induced writhing in mice.

Treatment groups	Dose (mg/kg)	Number of writhes		Percentage reduction (%)
		Mean ± SEM		
PS	0.25 mL	28.13	3.92	

*C. orbiculata*	100	13.83*	3.17	51
	200	9.20**	3.04	67
	400	6.75***	1.97	76

Paracetamol	300	2.10***	0.24	93

DMSO	0.25 mL	29.04	2.73	0

*P < 0.025, **P < 0.005, ***P < 0.001 versus 3% acetic acid (0.20 mL, i.p.) control, ANOVA (*n* = 8). Writhes are expressed as number of counts per 20 minutes.

PS: physiological saline.

DMSO: dimethylsulfoxide.

**Table 2:** Effect of leaf methanol extract of *Cotyledon orbiculata* on hot-plate-induced nociception in mice.

Treatment groups	Dose (mg/kg)	Response time (s)				
		0 min	15 min	30 min	45 min	60 min
PS	0.25 mL	4.13 ± 0.13	6.63 ± 0.60	4.38 ± 0.78	3.38 ± 0.68	2.75 ± 0.31

*C. orbiculata*	100	6.63 ± 0.92	10.25 ± 1.46	11.75** ± 1.07	7.13 ± 1.27	5.13 ± 0.08
	200	6.63 ± 0.85	7.38 ± 1.08	12.38^+^ ± 1.43	6.88 ± 1.57	6.25 ± 1.45
	400	5.25 ± 0.47	19.13* ± 5.01	26.63^++^ ± 3.35	22.63^+^ ± 3.41	24.5^++^ ± 2.55

Morphine	10	3.38 ± 0.64	26.63^++^ ± 4.83	36.50^++^ ± 6.55	22.88^++^ ± 2.93	16.63^++^ ± 2.07

DMSO	0.25 mL	5.00 ± 0.76	6.13 ± 0.99	3.88 ± 0.38	4.75 ± 0.86	4.50 ± 0.91

*P < 0.05, **P < 0.025, ^+^ *P* < 0.02, ^++^ *P* < 0.001 versus physiological saline control, ANOVA (*n* = 8). The response time in seconds was expressed as Mean ± SEM.

PS: physiological saline.

DMSO: dimethylsulfoxide.

**Table 3:** Effect of leaf methanol extract of Cotyledon orbiculata on carrageenan-induced oedema in the right hind paw of rat.

										
Treatment group	Dose (mg/kg)	Paw volume (mL) (Mean ± SEM)								
		0	30	60	90	120	150	180	210	240 (min)
UR	—	0.11 ± 0.01	0.12 ± 0.08	0.10 ± 0.03	0.09 ± 0.01	0.11 ± 0.04	0.11 ± 0.05	0.09 ± 0.07	0.10 ± 0.03	0.09 ± 0.05

PS	0.25 mL	0.09 ± 0.04	0.35 ± 0.05	0.48 ± 0.03	0.52 ± 0.02	0.61 ± 0.04	0.68 ± 0.02	0.72 ± 0.01	0.69 ± 0.05	0.69 ± 0.03

*C. orbiculata*	50	0.09 ± 0.01	0.29 ± 0.01	0.34* ± 0.01	0.41* ± 0.07	0.50* ± 0.02	0.53* ± 0.01	0.61* ± 0.04	0.59* ± 0.05	0.58* ± 0.03
	100	0.08 ± 0.05	0.26* ± 0.01	0.31** ± 0.04	0.37** ± 0.01	0.38^+^ ± 0.02	0.36^+^ ± 0.03	0.36^+^ ± 0.02	0.33^+^ ± 0.01	0.36^+^ ± 0.04
	200	0.11 ± 0.03	0.22* ± 0.02	0.26^+^ ± 0.01	0.27^+^ ± 0.01	0.28^+^ ± 0.04	0.31^+^ ± 0.02	0.30 ± 0.02	0.20^+^ ± 0.01	0.30^+^ ± 0.01
	400	0.10 ± 0.04	0.19* ± 0.02	0.22^+^ ± 0.01	0.21^+^ ± 0.03	0.21^+^ ± 0.02	0.20^+^ ± 0.04	0.22^+^ ± 0.01	0.19^+^ ± 0.01	0.19^+^ ± 0.02

Indomethacin	10	0.11 ± 0.02	0.14^+^ ± 0.02	0.18^+^ ± 0.04	0.20^+^ ± 0.06	0.19^+^ ± 0.03	0.18^+^ ± 0.01	0.17^+^ ± 0.02	0.16^+^ ± 0.03	0.15^+^ ± 0.04

DMSO	0.25 mL	0.11 ± 0.03	0.36 ± 0.06	0.44 ± 0.04	0.53 ± 0.07	0.64 ± 0.03	0.66 ± 0.04	0.70 ± 0.02	0.69 ± 0.03	0.67 ± 0.01

*P < 0.05, **P < 0.025, ^+^ *P* < 0.001 versus physiological saline control, ANOVA (*n* = 8).

UR: untreated rats.

PS: physiological saline.

DMSO: dimethylsuloxide.
